# Online Estimation of the Current Ripple on a Saturating Eerrite-Core Inductor in a Boost Converter

**DOI:** 10.3390/s20102921

**Published:** 2020-05-21

**Authors:** Matteo Lodi, Alberto Oliveri

**Affiliations:** Department of Electrical, Electronic, Telecommunications Engineering and Naval Architecture, University of Genoa, Via Opera Pia 11a, I-16145 Genova, Italy; alberto.oliveri@unige.it

**Keywords:** switch-mode power supply, ferrite-core inductor, magnetic saturation, nonlinear observer, microcontroller

## Abstract

In this paper, a nonlinear observer is proposed for the estimation of the current ripple in a ferrite-core inductor working in partial saturation, mounted on a boost converter. The estimator is based on a recently proposed nonlinear inductance model, which expresses the inductance as a function of the inductor current, taking into account also the non-negligible effects of the core temperature. The proposed observer is implemented on a low-cost microcontroller and tested, both offline and online, on a real boost converter with different operating conditions. The offline tests show a satisfactory estimation accuracy both during the electrical (fast) and thermal (slow) transients. Due to the high microcontroller latency, some delays and inaccuracies occur during electrical transients in the online tests. This work suggests that, in order to exploit the observer for control purposes, the target architecture should be a high-performance microcontroller, a system-on-chip, or a field programmable gate array, where parallelism can be exploited to speed-up the computations. The proposed implementation can be instead suitable for switch-mode power supply (SMPS) monitoring purposes.

## 1. Introduction

Switch-mode power supplies (SMPSs) are employed in all modern devices and systems using electrical energy: in consumer, industrial, aerospace, automotive, lighting, and other areas. Their use is expected to grow due to the increase of more electric vehicles in all transportation areas. SMPSs periodically store and release electrical energy, connecting power inductors alternately to the main energy source and to the load, through semiconductor devices operating as switches, typically driven by a pulse-width-modulation (PWM) signal.

The frequency and duty cycle of the PWM signal are imposed by a control system, such that the converter output voltage is maintained at a reference value and the current flowing through the inductor does not exceed safety limits, independently of variations in the input voltage and load current. The simplest control strategy is the voltage mode control, where the difference between the output voltage and the reference value is used as input for the controller, e.g., a proportional-integral regulator. The main drawback of this approach is that it has a slow response to load current variations, and good closed-loop performances are difficult to achieve [1,2].

With current mode control, the voltage error signal provides a current reference, which is compared to the inductor current in order to obtain the control signal. A dual loop control is therefore performed, which allows for faster transient response and (limited) over-current protection [3]. Current mode control of course requires measurements of the inductor current, which can be basically performed through shunt resistors, mirroring circuits, or Hall effect sensors [4,5]. The shunt resistor is very simple, but it has a low accuracy, modifies the circuit, and increases the power loss. Mirroring circuits are sensitive to electromagnetic interference and also exhibit a low accuracy. On the other hand, Hall effect sensors do not have these disadvantages, but they are very expensive. In any case, all these current sensing techniques add noise to the system and increase the overall converter power consumption, size, and cost [6].

Sensorless current mode control [7] has therefore gained popularity (see [2] and the references therein) as it allows improving the reliability of the system, the miniaturization of the device, its efficiency, and its cost. Of course, an observer is required, which estimates the inductor current based on measurements of other electrical variables. Many approaches have been proposed in the literature [8,9,10,11,12,13]. In all of these works, the inductor is assumed to work in its linear region, where its inductance is constant. In this case, the converter dynamics is linear for each configuration of the switches, and classical bilinear averaged SMPS models can be exploited for the observer design.

Recent studies [14,15,16] showed that it was possible to design smaller and lighter SMPS by exploiting ferrite-core inductors operating in partial saturation. In this situation, the inductance drops as the inductor current increases, with a strong dependence on the core temperature (at higher temperatures, the inductance starts dropping for lower current values). Several nonlinear behavioral models have been therefore proposed (see [17] for a survey), which take into account these nonlinear phenomena. In [18,19], in particular, the inductance was modeled as an arctangent function of the inductor current, which translates towards the left as the temperature increases. A piecewise-affine (PWA) version of the same model was proposed in [20] and used in [21], which allows computing analytically the inductor current, based on the inductor voltage. With a nonlinear inductor model, the overall SMPS is nonlinear for each switch configuration, and an averaged model cannot be used.

In this paper, we propose a nonlinear observer, based on the model proposed in [20], able to estimate analytically the inductor current waveform, based on easily available measurements of the SMPS input and output voltage and of the load current. In order to spare computation time, only the minimum, maximum, and average current within a PWM period are actually estimated, which are the most relevant quantities for control and monitoring purposes [22,23,24]. A disturbance term is also estimated, based on the measurements of the SMPS output voltage. This allows partially compensating for model parameters’ uncertainties. The load current has a much slower dynamics with respect to the inductor current and can be acquired with a lower accuracy as it is not directly used for control purposes. For this reason, a simple shunt resistor can be exploited. However, the measurement of the inductor current is necessary to train the inductor model. To this aim, a laboratory SMPS is equipped with a Hall effect current sensor, which will not be present on the SMPSs operating in the real applications.

The proposed estimator is tested both offline and online, based on measurements performed on a real boost converter. Different operating conditions (PWM frequency, duty cycle, and output current) are applied to the SMPS, such that the inductor works both in its linear region and in saturation. The relative error at steady-state and during slow thermal transients is below 10%.

The online tests are performed by implementing the proposed observer on a low-cost microcontroller. In this case, some inaccuracies and delays can be observed during fast electrical transients, mainly due to the high microcontroller latency (much higher than the PWM period). This suggests that, in order to be successfully applied for sensorless current mode control, the proposed observer should be implemented on a high-performance microcontroller, a system-on-chip (SoC), or a field programmable gate array (FPGA). The proposed implementation could be instead exploited for monitoring purposes, as the observer is able to sense a potentially dangerous increase of the inductor current in a few milliseconds.

A preliminary simpler observer based on the model in [20] was proposed in [21]; the novelties of this work with respect to [21] are:

- A disturbance term is added to the observer, estimated based on measurements of the output voltage, which makes the observer more robust with respect to model parameters’ uncertainties. This is of paramount importance in practice, because model uncertainties (due for example to the tolerances of the components) are always present;
- The estimated quantities are computed analytically; in [21], an iterative procedure based on the Euler method was used;
- Due to the analytical formulation, measurements can be acquired every 200 μs, whereas a sampling period of 10 ms was used in [21] in the same experiments, which would cause severe delays during electrical transients, preventing the application of the observer even for monitoring purposes;
- Tests during fast electrical transients are performed.

## 2. Materials and Methods

**Figure 1:**
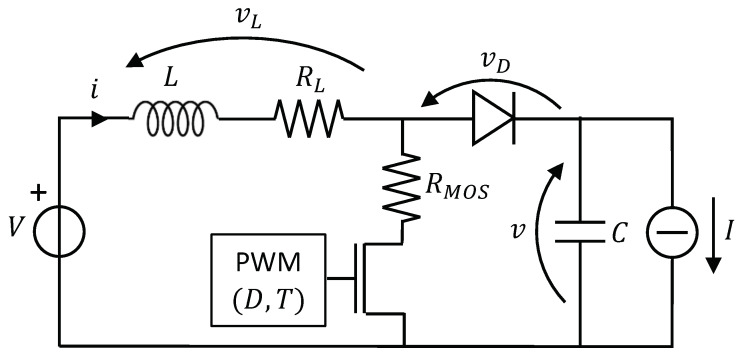
Schematic of a boost converter.

Consider the boost converter shown in Figure 1. The operating conditions are the input voltage *V*, the output current *I*, the period *T* (frequency $F=\frac{1}{T}$), and duty cycle *D* of the PWM signal, which drives the MOS transistor operating as a switch. Variables *i* and *v* are the inductor current and capacitor voltage (output voltage), respectively, whereas $v_{D}$ is the voltage drop across the diode and $v_{L}$ the voltage drop across the inductor, including its parasitic series resistance $R_{L}$. We also indicate with $R_{MOS}$ the parasitic resistance of the transistor when it works as a closed switch (ON resistance).

**Figure 2:**
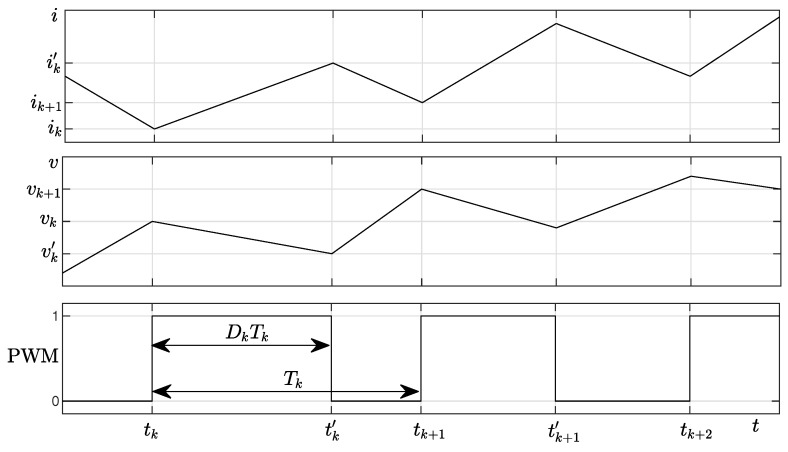
Typical waveforms during electrical transients for *i* (top panel), *v* (middle panel), and the PWM driving signal (bottom panel).

Figure 2 shows typical waveforms during electrical transients for *i* (top panel), *v* (middle panel), and the PWM driving signal (bottom panel). We denote with $t_{k}$ the starting instant of the $k^{\mathrm{th}}$ PWM cycle, with period $T_{k}$ (frequency $F_{k}=\frac{1}{T_{k}}$) and duty cycle $D_{k}$. We also define $i_{k}=i\left(t_{k}\right)$, $v_{k}=v\left(t_{k}\right)$, $t_{k}^{\prime }=t_{k}+D_{k}T_{k}$, $i_{k}^{\prime }=i\left(t_{k}^{\prime }\right)$, $v_{k}^{\prime }=v\left(t_{k}^{\prime }\right)$. We assume that *V*, *I*, and *v* can be always measured, whereas *i* and $v_{L}$ are measured only on a laboratory converter for training the inductance model.

**Figure 3:**
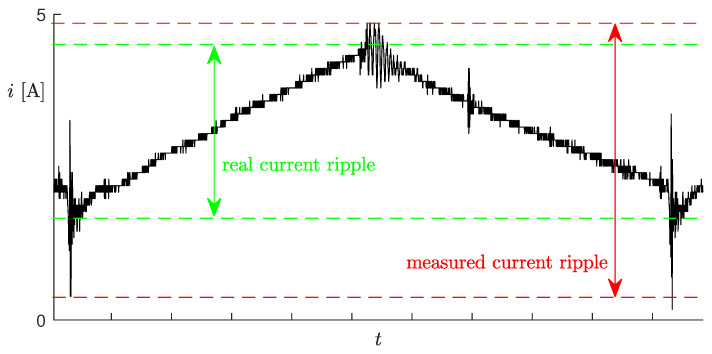
Unfiltered inductor current measurement with the indication of the real current ripple (green) and the wrong current ripple, which could be obtained based on the measurements.

The online measurement of the voltage $v_{L}$ would not be a big issue, but it is actually unnecessary, whereas the measurement of the inductor current is unpractical, as explained in the Introduction of this paper. Moreover, Figure 3 shows the unfiltered inductor current measured through a Hall effect sensor (an LEM LTS 6-NP). Notice the large oscillations in correspondence to the PWM switches, which could lead to the wrong ripple estimations.

In order to model the inductor properly also when it works in partial saturation, we used the nonlinear behavioral model proposed in [20], where the inductance *L* is expressed as a piecewise-affine function of the inductor current, in particular:

(1) $$L\left(i-J\right)=a_{h}\cdot \left(i-J\right)+b_{h}\;\mathrm{if}X_{h}\leq i-J<X_{h+1}$$

where $X_{0}$ and $X_{m}$ are the domain boundaries and $X_{h}$ (h=1,…,m−1) are the knee points of the PWA function, with $X_{h+1}>X_{h}$. It was shown in [15,18] that the *L* vs. *i* curve shifts towards the left as the core temperature increases. This phenomenon is reproduced by the behavioral state variable $J=J(p_{k},t)$, which does not directly represent a physical quantity and takes into account the effect of core temperature variations [19]. State *J* satisfies the following first-order equation:

(2) $$\tau \frac{dJ}{dt}=\alpha p_{k}+\beta -J$$

for $t\in [t_{k},\;t_{k+1})$, $p_{k}$ being the average inductor power loss within the $k^{\mathrm{th}}$ PWM cycle and α and β fitting parameters.

A very simple model that relates the average power loss to the SMPS operating conditions was successfully used in [19] and is also exploited in this paper. We express the estimated average power loss as:

(3) $${\tilde{p}}_{k}=\left(\gamma +D_{k}\delta \right)i_{RMS,k}^{2}$$

where the root mean squared current on the $k^{\mathrm{th}}$ PWM cycle is defined as:

(4) $$i_{RMS,k}=\sqrt{\frac{1}{T_{k}}\int_{t_{k}}^{t_{k+1}}i^{2}\left(t\right)dt}$$

Parameters γ and δ must be fitted to experimental measurements.

By referring to the circuit shown in Figure 1, the boost converter state equations in the $k^{\mathrm{th}}$ PWM cycle are:

fortk≤t<tk′:(5)didt=V−(RL+RMOS)i+ηL(i−J)(6)dvdt=−IC

fortk′≤t<tk+1:(7)didt=V−vD−RLi−v+ηL(i−J)(8)dvdt=i−IC

where η is a disturbance term that takes into account the modeling errors, such as the uncertainties on parasitic resistances. This unmeasurable quantity is estimated through the proposed observer.

### 2.1. Model Fitting

The power loss model depends on parameters γ and δ. Given the measurements of the inductor voltage $v_{L}$ and current *i*, the average power loss within the $k^{\mathrm{th}}$ PWM period can be computed as:

(9) $$p_{k}=\frac{1}{T_{k}}\int_{t_{k}}^{t_{k+1}}v_{L}\left(t\right)i\left(t\right)dt$$

The parameters γ and δ can be therefore obtained by solving the following quadratic programming (QP) optimization problem:

(10) $$\min_{\gamma ,\delta }\sum_{k\in K}{\left[p_{k}-{\tilde{p}}_{k}\left(\gamma ,\delta \right)\right]}^{2}$$

where K is the set of indices denoting the PWM cycles chosen for training the power loss model.

The inductance model depends on parameter vector $x=[X_{1},\ldots ,X_{m-1},L\left(X_{1}\right),\ldots ,L\left(X_{m-1}\right),\alpha ,\beta ,\tau ,R_{L}]$; the number of knee points *m* and the domain boundaries $X_{0}$ and $X_{m}$ are fixed a priori. As shown in [19], *x* can be obtained by solving the following nonlinear optimization problem:

(11) $$\min_{x}\sum_{t\in T}{\left[i\left(t\right)-\tilde{ı}\left(t;x\right)\right]}^{2}$$

where T is the set of times used for training the model, i(t) are samples of the measured inductor current, whereas $\tilde{ı}\left(t;x\right)$ are the current values obtained by simulating the boost converter (through Equations (1)–(8)) at the same time instants, by applying the same operating conditions. Problem (10) is a quadratic programming (QP) problem, with respect to parameters γ and δ; therefore, it can be easily solved with any QP algorithm. Problem (11) is instead a nonlinear programming (NLP) problem, which can be solved, e.g., with evolutionary algorithms, simulated annealing, and direct search methods.

### 2.2. Nonlinear Observer

We indicate with a “hat” the estimated variables provided by the proposed observer. The input voltage and output current have a much slower dynamics with respect to *i* and *v*; therefore, we assume that $V=V_{k}$ and $I=I_{k}$, within the $k^{\mathrm{th}}$ PWM cycle. Moreover, it was shown in [19] that also the dynamics of *J* is much slower than the dynamics of *i* and *v*, as it is related to the temperature. We can therefore assume that $J(p_{k},t)$ assumes the constant value $J_{k}=J\left(p_{k},t_{k}\right)$ within the $k^{\mathrm{th}}$ PWM period. We further assume $\eta =\eta_{k}$ within the $k^{\mathrm{th}}$ PWM period.

At the beginning of the $k^{\mathrm{th}}$ PWM cycle, the measurements $V_{k}$, $I_{k}$, and $v_{k}$ are available, as well as values ${\hat{v}}_{k}$, ${\hat{ı}}_{k}$, $J_{k}$, $\eta_{k-1}$, ${\bar{ı}}_{k-1}^{\prime }$, and ${\bar{ı}}_{k-1}^{\prime \prime }$, where:

(12) $${\bar{ı}}_{k-1}^{\prime }≜\frac{1}{D_{k-1}T_{k-1}}\int_{t_{k-1}}^{t_{k-1}^{\prime }}\hat{ı}\left(t\right)dt,\;{\bar{ı}}_{k-1}^{\prime \prime }≜\frac{1}{\left(1-D_{k-1}\right)T_{k-1}}\int_{t_{k-1}^{\prime }}^{t_{k}}\hat{ı}\left(t\right)dt$$

The aim of the proposed observer is to estimate ${\hat{v}}_{k+1}$, ${\hat{ı}}_{k}^{\prime }$, ${\hat{ı}}_{k+1}$, $J_{k+1}$, $\eta_{k}$, ${\bar{ı}}_{k}^{\prime }$, and ${\bar{ı}}_{k}^{\prime \prime }$. Based on these quantities, it is possible to obtain the estimated inductor current ripple and the mean current.

- Estimation of $\eta_{k}$:
  The disturbance can be estimated based on the measurements of the output voltage $v_{k}$:
  (13) $$\eta_{k}=\eta_{k-1}+K\left(v_{k}-{\hat{v}}_{k}\right)$$
  where *K* is a positive gain. With this strategy, η is corrected until the estimated voltage coincides with the measured one.
- Estimation of ${\hat{ı}}_{k}^{\prime }$:
  By integrating Equation (5) between $t_{k}$ and $t_{k}^{\prime }$, it is possible to obtain:
  (14) $$\int_{t_{k}}^{t_{k}^{\prime }}L\left(i-J_{k}\right)di=\int_{t_{k}}^{t_{k}^{\prime }}\left(V_{k}+\eta_{k}\right)dt-\left(R_{L}+R_{MOS}\right)\int_{t_{k}}^{t_{k}^{\prime }}idt$$
  The last integral is actually equal to $D_{k}T_{k}{\bar{ı}}_{k}^{\prime }$, where ${\bar{ı}}_{k}^{\prime }$ is not known. However, it is reasonable to assume that the current waveform does not change too much between the $k-1^{\mathrm{th}}$ and $k^{\mathrm{th}}$ PWM period, except for the case when the operating conditions are suddenly changed. For this reason, we will assume ${\bar{ı}}_{k}^{\prime }≊{\bar{ı}}_{k-1}^{\prime }$, which is instead known. The following initial value problem can be therefore defined:
  (15) $$\left\{\begin{array}{c} \frac{d\hat{ı}}{dt}=\frac{V_{k}-\left(R_{L}+R_{MOS}\right){\bar{ı}}_{k-1}^{\prime }+\eta_{k}}{L(\hat{ı}-J_{k})}≜\frac{W_{k}}{L(\hat{ı}-J_{k})}\;\left(W_{k}\mathrm{is}a\mathrm{constant}\right)\\ \hat{ı}\left(t_{k}\right)={\hat{ı}}_{k} \end{array}\right.$$
  which can be solved analytically (see Appendix A) in order to obtain ${\hat{ı}}_{k}^{\prime }$. As shown in Appendix A, we can also obtain analytically $\int_{t_{k}}^{t_{k}^{\prime }}\hat{ı}\left(t\right)dt$ and $\int_{t_{k}}^{t_{k}^{\prime }}{\hat{ı}}^{2}\left(t\right)dt$.
- Estimation of ${\hat{ı}}_{k+1}$:
  By integrating Equation (6) between $t_{k}$ and $t_{k}^{\prime }$, it is possible to obtain:
  (16) $${\hat{v}}_{k}^{\prime }={\hat{v}}_{k}-D_{k}T_{k}\frac{I_{k}}{C}$$
  and by integrating Equation (18) between $t_{k}^{\prime }$ and $t_{k+1}$, we obtain:
  (17) $$\int_{t_{k}^{\prime }}^{t_{k+1}}L\left(i-J_{k}\right)di=\int_{t_{k}^{\prime }}^{t_{k+1}}\left(V_{k}-v_{D}+\eta_{k}\right)dt-R_{L}\int_{t_{k}^{\prime }}^{t_{k+1}}idt-\int_{t_{k}^{\prime }}^{t_{k+1}}vdt$$
  Here, $\int_{t_{k}^{\prime }}^{t_{k+1}}idt=\left(1-D_{k}\right)T_{k}{\bar{ı}}_{k}^{\prime \prime }$. We assume $\int_{t_{k}^{\prime }}^{t_{k+1}}vdt≊0.5\left({\hat{v}}_{k}+{\hat{v}}_{k}^{\prime }\right)$ and ${\bar{ı}}_{k}^{\prime \prime }≊{\bar{ı}}_{k-1}^{\prime \prime }$.
  The following initial value problem can be therefore defined:
  (18) $$\left\{\begin{array}{c} \frac{d\hat{ı}}{dt}=\frac{V_{k}-v_{D}-R_{L}{\bar{ı}}_{k-1}^{\prime \prime }-0.5\left({\hat{v}}_{k}+{\hat{v}}_{k}^{\prime }\right)+\eta_{k}}{L(\hat{ı}-J_{k})}≜\frac{W_{k}^{\prime }}{L(\hat{ı}-J_{k})}\;\left(W_{k}^{\prime }\mathrm{is}a\mathrm{constant}\right)\\ i\left(t_{k}^{\prime }\right)={\hat{ı}}_{k}^{\prime } \end{array}\right.$$
  which can be solved analytically (see Appendix A) in order to obtain ${\hat{ı}}_{k+1}$. As shown in Appendix A, we also obtain analytically $\int_{t_{k}^{\prime }}^{t_{k+1}}\hat{ı}\left(t\right)dt$ and $\int_{t_{k}^{\prime }}^{t_{k+1}}{\hat{ı}}^{2}\left(t\right)dt$.
- Estimation of ${\hat{v}}_{k+1}$:
  By integrating Equation (8) between $t_{k}^{\prime }$ and $t_{k+1}$, we obtain:
  (19) $$\begin{array}{cc} {\hat{v}}_{k+1} & ={\hat{v}}_{k}^{\prime }+\frac{1}{C}\int_{t_{k}^{\prime }}^{t_{k+1}}\left[\hat{ı}\left(t\right)-I_{k}\right]dt= \end{array}$$
  (20) $$\begin{array}{cc} \; & ={\hat{v}}_{k}^{\prime }+\frac{1}{C}\int_{t_{k}^{\prime }}^{t_{k+1}}\hat{ı}\left(t\right)dt-\frac{\left(1-D_{k}\right)T_{k}}{C}I_{k}= \end{array}$$
  (21) $$\begin{array}{cc} \; & ={\hat{v}}_{k}+\frac{1}{C}\int_{t_{k}^{\prime }}^{t_{k+1}}\hat{ı}\left(t\right)dt-\frac{T_{k}}{C}I_{k} \end{array}$$
  where all terms have already been computed in the previous steps.
- Estimation of $J_{k+1}$:
  By applying the forward Euler method to Equation (2), we obtain:
  (22) $$J_{k+1}=\frac{\tau -T_{k}}{\tau }J_{k}+\frac{T_{k}\left(\alpha p_{k}+\beta \right)}{\tau }$$
  where the power loss model is exploited to compute:
  (23) $$p_{k}=\left(\gamma +D_{k}\delta \right)i_{RMS,k}^{2}$$
  where:
  (24) $$i_{RMS,k}^{2}=\frac{1}{T_{k}}\int_{t_{k}}^{t_{k+1}}i^{2}dt=\frac{1}{T_{k}}\int_{t_{k}}^{t_{k}^{\prime }}i^{2}dt+\frac{1}{T_{k}}\int_{t_{k}^{\prime }}^{t_{k+1}}i^{2}dt$$
  and the integrals have already been computed in the previous steps.
- Estimation of ${\bar{ı}}_{k}^{\prime }$ and ${\bar{ı}}_{k}^{\prime \prime }$:
  The estimated mean current values in intervals $\left[t_{k},\;t_{k}^{\prime }\right]$ and $\left[t_{k}^{\prime },\;t_{k+1}\right]$ can be computed as:
  (25)ı¯k′=1DkTk∫tktk′idt(26)ı¯k″=1(1−Dk)Tk∫tk′tk+1idt
- Estimation of the current ripple and mean current:
  Finally, the inductor current ripple $\Delta_{k}$ and mean current ${\bar{ı}}_{k}$ in interval $\left[t_{k},\;t_{k+1}\right]$ can be estimated as:
  (27)Δk=ı^k′−ı^k(28)ı¯k=1Tk∫tktk+1idt=1Tk∫tktk′idt+1Tk∫tk′tk+1idt

### 2.3. Initial Conditions

The initial conditions (at k=0) for the estimated variables can be reasonably set by performing the following assumptions:

- the converter is ideal (there are no power losses);
- the inductor works in its linear region.

These assumptions are quite restrictive; however, the initial conditions do not need to be really accurate, as the observer is able to converge to the correct values in a few iterations. This is just a strategy to set them close to the correct value.

The output voltage is measured; therefore:

(29) $${\hat{v}}_{0}=v_{0}$$

For an ideal converter, the average input power coincides with the output power, i.e., $\int_{t_{k}}^{t_{k+1}}Vidt=\int_{t_{k}}^{t_{k+1}}vIdt$. By assuming *V*, *v*, and *I* constant, we can derive:

(30) $${\bar{ı}}_{0}^{\prime }={\bar{ı}}_{0}^{\prime \prime }=\frac{v_{0}I_{0}}{V_{0}}$$

If the inductor is assumed to work in its linear region, then the ripple can be computed as:

(31) $$\Delta_{0}=\frac{V_{0}D_{0}T_{0}}{L_{nom}}$$

which allows setting:

(32) $$\begin{array}{cc} {\hat{ı}}_{0} & ={\bar{ı}}_{0}^{\prime }-0.5\Delta_{0} \end{array}$$

(33) $$\begin{array}{cc} {\hat{ı}}_{0}^{\prime } & ={\bar{ı}}_{0}^{\prime }+0.5\Delta_{0} \end{array}$$

If we assume that the inductor current is a perfect triangular wave (which is the case, with the considered assumptions), we can compute its root mean squared value as:

(34) $${\hat{ı}}_{RMS,0}^{2}={\left({\bar{ı}}_{0}^{\prime }\right)}^{2}+\frac{{\left(0.5\Delta_{0}\right)}^{2}}{3}$$

which allows setting the initial power loss as:

(35) $${\hat{p}}_{0}=\left(\gamma +D_{0}\delta \right){\hat{ı}}_{RMS,0}^{2}$$

### 2.4. Experimental Setup

The embedded estimator was implemented on a STM32F4 microcontroller and tested on a boost converter composed of a bank of capacitors with total capacitance C = 330 μF, an MSS1038T-103 inductor with a nominal inductance $L_{nom}$ = 10 μH, and a switch realized through a IXTP230N075T2 MOS transistor with $R_{MOS}$ = 250 mΩ. A common diode with voltage drop $v_{D}$ = 0.7 V was exploited. The load current *I* was imposed through a PEL 3031E DC electronic load. The input voltage *V* was applied through a BREMI BRS 55 power source, whereas the driving PWM signal was provided to the MOS gate by means of the same STM32F4 microcontroller, through an IX6R11S3 MOS driver. A RIGOL DS1000Z oscilloscope with 10 bit resolution was used to sense the inductor current *i* (through an LEM LTS 6-NP Hall effect transducer), which was the benchmark to evaluate the accuracy of the proposed estimation method. This setup allowed applying different working conditions to the converter. In this work, we set all the possible combinations of *V*, *I*, *F*, and *D* shown in Table 1 to train the inductance and power loss models. For the highest current values, the inductor worked in partial saturation. The power loss and inductance model parameters were identified starting from the experimental data by solving optimization problems (10) with MATLAB routine quadprog and (11) through a mesh adaptive direct search algorithm [25], and the resulting parameters are listed in Table 2. In order to speed up the computation on the microcontroller, we fixed the knee points of the inductance PWA function equally spaced in the interval [−20,20]A, in particular $X_{0}=-20$A, $X_{m}=20$A, and $X_{k}=X_{k-1}+\frac{X_{m}-X_{0}}{m}$, k=1,…,m−1. The number of knee points was chosen as m=13, because a larger number would not improve significantly the modeling accuracy. The estimator gain was set heuristically to K=0.01.

### 2.5. Microcontroller Implementation

**Figure 4:**
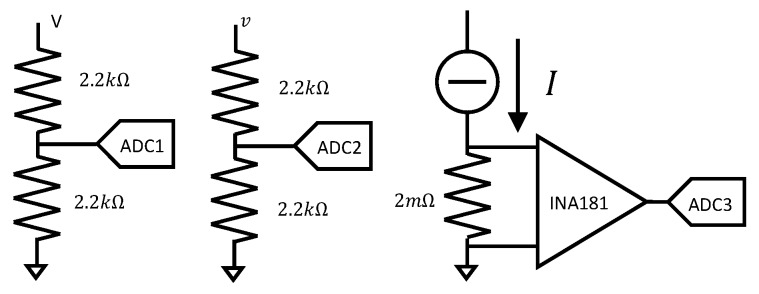
Signal conditioning circuit for online data acquisition.

The microcontroller generated the PWM signal, and therefore, it imposed the PWM period $T_{k}$ and duty cycle $D_{k}$. The input voltage $V_{k}$, the load current $I_{k}$, and the output voltage $v_{k}$ were acquired at the beginning of each PWM cycle through three analog-to-digital converters (ADCs) with 12 bit resolution and were stored in the microcontroller RAM through the integrated direct-memory-access (DMA) interface. The sampling time was therefore variable as it was equal to $T_{k}$. The circuits used to scale the measured quantities to the ADC range ([0,3]V) are shown in Figure 4. In particular, the output current was converted into a voltage through a 2mΩ resistor and a Texas Instruments INA 181 current sense amplifier (right panel in Figure 4), whereas voltages *v* and *V* were scaled through voltage dividers (left and middle panels).

As shown in Appendix A, many terms necessary for the estimation could be pre-computed offline, and a uniform distribution of the PWA knee points was used, which allowed speeding up the computation. The computation time was not constant, as it depended on how many regions of the PWA function being explored; we measured a maximum latency of about $T_{c}$ = 200 μs. The sampling period was selected as the first multiple of the PWM frequency higher than $T_{c}$. For the PWM frequencies used in this paper (50, 70, and 100 kHz), the sampling frequency was 5kHz. The estimated current values ${\hat{ı}}_{k}$ and ${\hat{ı}}_{k}^{\prime }$ were provided (every 200 μs) though two digital-to-analog converters. A single precision floating point representation was adopted in the microcontroller.

## 3. Results

For these tests, a different sample of the MSS1038T-103 inductor was used, in order also to assess the robustness of the inductance model with respect to different components. The Hall effect current probe was used here to measure the inductor current only in order to compare it with the estimated one, but it was not necessary for the estimation. Tests during fast electrical transients and slow thermal transients were performed. The first tests were performed both offline, in order to assess the accuracy of the estimator if computation delays were neglected, and online, where the estimator ran on the microcontroller together with the boost converter. The tests on thermal transients were only performed online.

### 3.1. Electrical Transient

#### 3.1.1. Offline Tests

**Figure 5:**
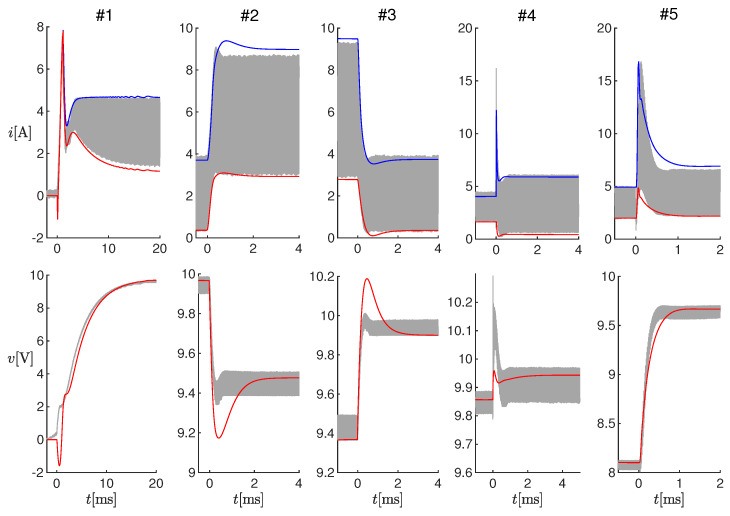
Results of offline estimations on Tests #1-#5 (see Table 3). Top panels: measured current (gray curves) and estimated values ${\hat{ı}}_{k}$ (red curves) and ${\hat{ı}}_{k}^{\prime }$ (blue curves). Bottom panel: measured output voltage (gray curves) and estimated value ${\hat{v}}_{k}$ (red curves).

For these offline tests, measurements of the inductor current, input voltage, and output voltage were obtained in correspondence with five changes in the SMPS operating conditions, listed in Table 3. These time series, together with the operating conditions, were provided to the proposed observer with a sampling time equal to the PWM period. The latency was therefore assumed to be lower than the PWM period. Figure 5 shows the inductor current (top panels) and the output voltage (bottom panels) for each test. The gray curves are the measured values i(t) and v(t), filtered through a Savitzky–Golay smoothing filter with order one and a frame length of 200ns, whereas the red and blue curves in the top panels correspond to ${\hat{ı}}_{k}$ and ${\hat{ı}}_{k}^{\prime }$, respectively, and the red curves in the bottom panels correspond to ${\hat{v}}_{k}$.

**Figure 6:**
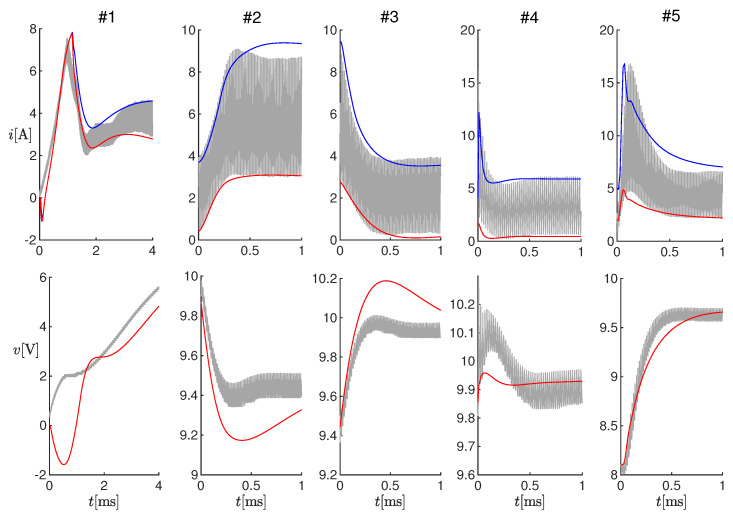
Enlargements of the panels in Figure 5.

Figure 6 shows enlargements of the panels in Figure 5, in correspondence to the changes in the operating conditions. It can be noticed that the estimated electrical transients were qualitatively similar to the measured ones. The differences were due to the model uncertainties, which were partially compensated at steady-state through variable η. In order to quantify the performance of the observer, we could compute the difference between the times when the estimated and measured currents reached their steady-state, and we obtained about 0ms for Test #1, 2ms for Tests #2-#4, and 0.7 ms for Test #5.

**Figure 7:**
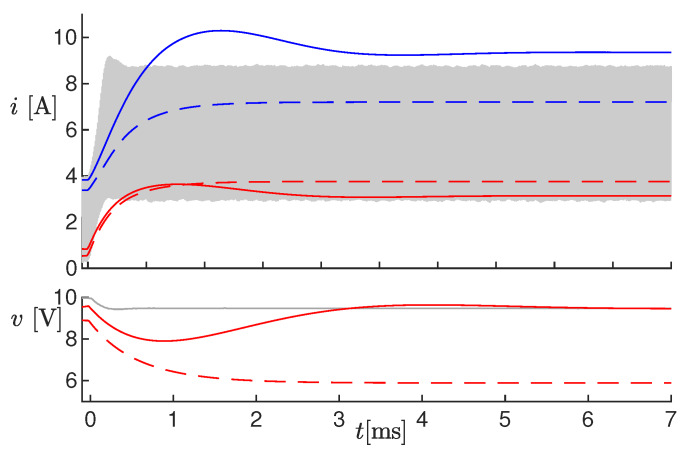
Results of Test #2 with parameter $R_{L}$ increased ten times. The dashed lines are the estimated values obtained with η=0.

In order to check the ability of the observer to compensate for errors in some parameters, we repeated again Test #2 with the inductor’s parasitic series resistance $R_{L}=350$ mΩ, ten times larger than the real value. The results are shown in Figure 7. The solid blue and red lines are the estimated values. Notice that the voltage converged to the real value, and ${\hat{ı}}_{k}^{\prime }$ exhibited a larger overshoot (compared to Figure 5), but got close to the real value at steady-state. This behavior was due to the correction term η. The dashed lines show indeed the same results with η=0, leading to completely wrong estimations also at steady-state.

#### 3.1.2. Online Tests

**Figure 8:**
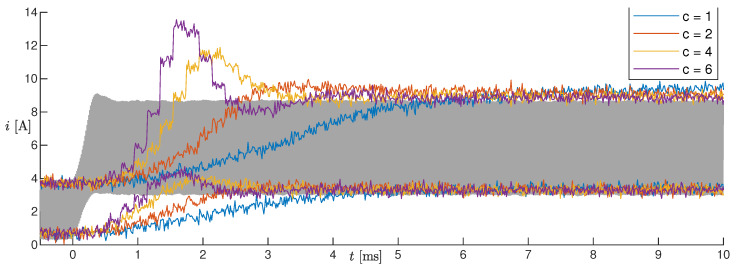
Results of Test #2 with different values of *c*.

For these tests, the observer was implemented on the microcontroller and operated online together with the SMPS. The tests on the five changes in the operating conditions listed in Table 3 were repeated, where the measurements were performed online by the microcontroller with a sampling period of 200 μs. The high microcontroller latency, which implied using a sampling period much larger than the PWM period, constituted actually a problem during fast electrical transients, as the estimated voltage and current transients resulted in being much slower (tens of milliseconds) than the real ones (see the blue lines in Figure 8). In order to compensate for this delay partially, we made the model used by the estimator faster by imposing its capacitance as $\hat{C}=\frac{C}{c}$, with c>0. Figure 8 shows the results of Test #2 obtained with different values of *c*, from one to six. Notice that with the original capacitance value (i.e., c=1), the estimated current did not even reach the steady-state value in 10 ms, whereas as *c* increased, the convergence speed also increased. By setting c>4, oscillations appeared in the estimation, which were not present in the measurements. For this reason, we chose c=4. We remark that scaling the capacitance had a negligible effect on the estimation at steady-state, as the capacitance mainly influenced the output voltage ripple, which we did not estimate.

**Figure 9:**
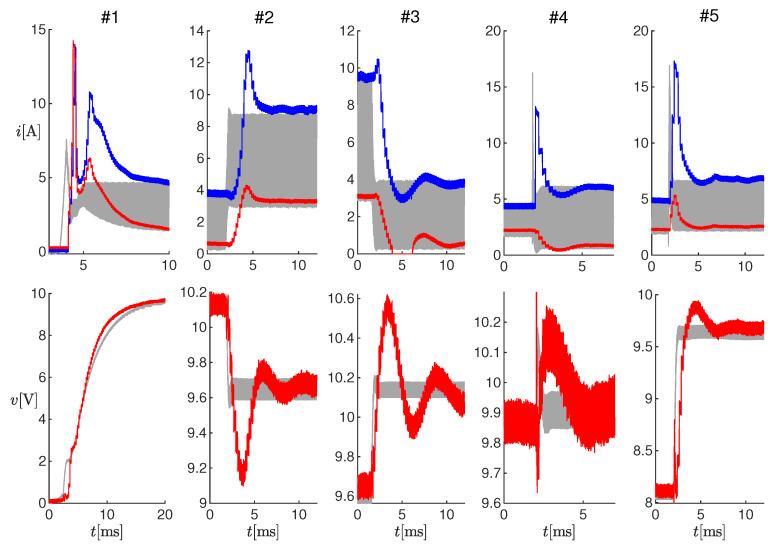
Results of online estimations on Tests #1-#5 (see Table 3). Top panels: measured current (gray curves) and estimated values ${\hat{ı}}_{k}$ (red curves) and ${\hat{ı}}_{k}^{\prime }$ (blue curves). Bottom panel: measured output voltage (gray curves) and estimated value ${\hat{v}}_{k}$ (red curves).

Figure 9 shows the results of the online tests with the scaled capacitance (c=4). The performance at steady-state was comparable with the one obtained in the offline tests, whereas the effects of the high latency, even if mitigated by the scaled capacitance, were clearly visible during the transients.

**Figure 10:**
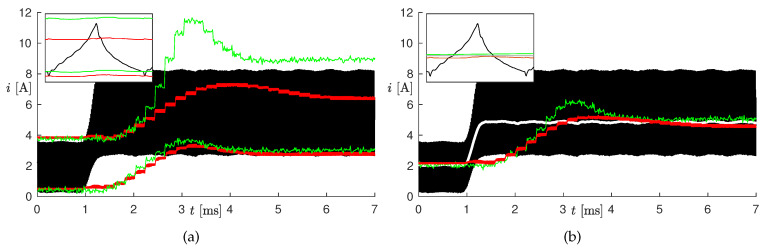
Inductor current on Test #2 with nonlinear (green curves) and constant (red curves) inductance. The black curve is the measured current and the gray curve in panel (**b**) its mean value over each PWM period. The green (red) curves in panel (**a**) are the minimum and maximum currents estimated with the nonlinear (constant) inductance. Panel (**b**) shows instead the mean estimated values.

In order to appreciate the benefits of using a nonlinear inductance model, we performed Test #2 by using a constant inductance $L=L_{nom}$ = 10 μH. The results are shown in Figure 10: the black curve is the measured current; the green curves are the estimated values with the nonlinear inductance (proposed observer); whereas the red curves are the estimated values with constant inductance. The left panels show values ${\hat{ı}}_{k}$ and ${\hat{ı}}_{k}^{\prime }$ (minimum and maximum current), whereas the right panels show the average current within each PWM period. Notice that, by using a constant inductance, the estimation was accurate for low currents (when the inductor worked in the linear region), but the ripple was strongly underestimated for high currents (when the inductor worked in saturation). The mean current was instead currently estimated at steady-state also with the constant inductance. This was not surprising, as the current ripple was mainly determined by the SMPS operating conditions and not by the inductance itself. The enlargement proved that the inductor was saturating, as the current was not a triangular wave [15]. The relative percent error on the ripple, after the change in the load current, was 4.85% with the nonlinear inductance and −27.5% with the constant inductance. This result was expected as the constant inductance model could not model the inductor behavior in saturation.

### 3.2. Thermal Transient

**Figure 11:**
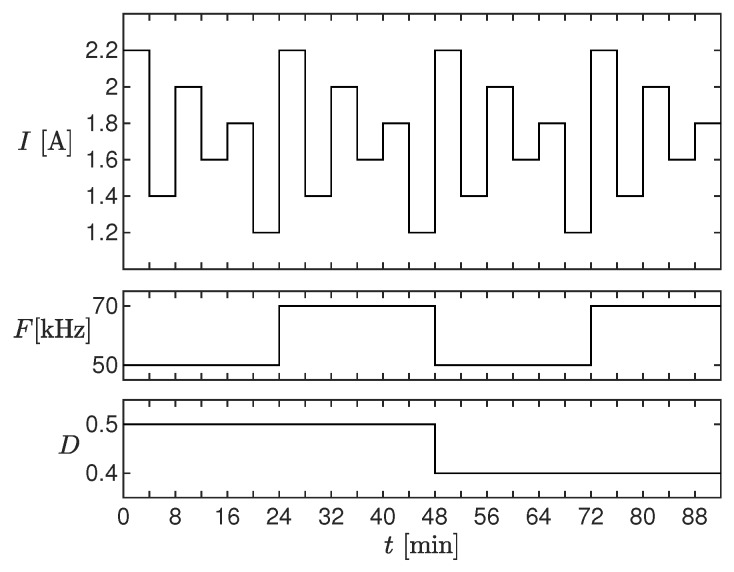
Operating conditions used for testing the estimator.

**Figure 12:**
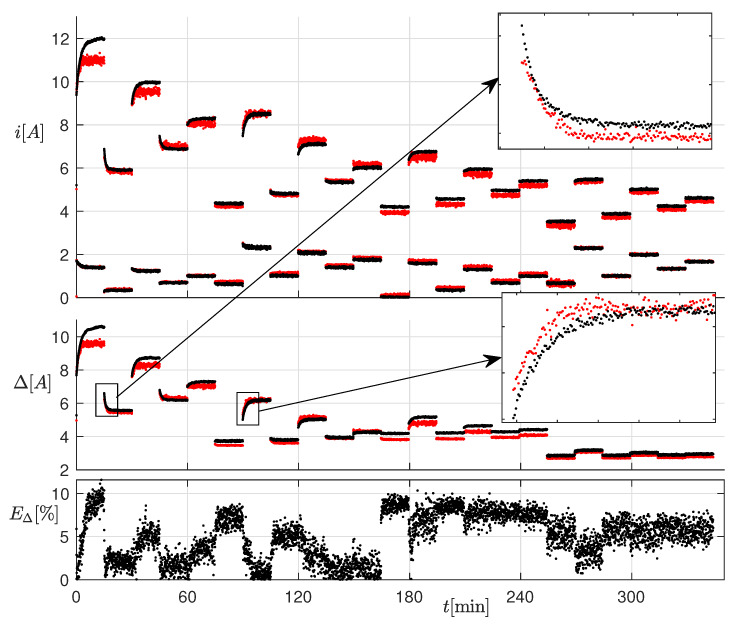
Top panel: measured (black) and estimated (red) current at electrical steady-state for all the considered operating conditions. Middle panel: measured (black) and estimated (red) current ripple. Bottom panel: relative percent error on the current ripple.

For this test, we set the operating conditions shown in Figure 11. Each operating condition was kept constant for 15 min, in order to appreciate the effects of temperature. The top panel of Figure 12 shows the measured (black) and estimated (red) current envelope at electrical steady-state for all the considered operating conditions. The current ripple is shown in the middle panel, whereas the relative percent error on the current ripple is shown in the bottom panel. The measurements were acquired every 4 s. Notice that the ripple error was below 10% in almost all the considered conditions, also those where the inductor worked in saturation. Moreover, as clearly visible in the enlargement, the slow ripple drift due to the temperature variation was correctly reproduced: this was due to the state variable *J*. This effect would not have been taken into account with the standard linear inductor model.

**Figure 13:**
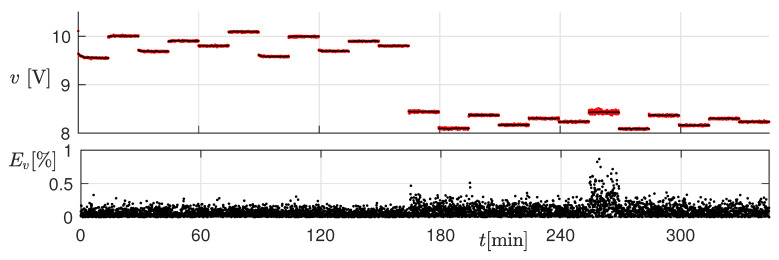
Top panel: measured (black) and estimated (red) output voltage at electrical steady-state for all the considered operating conditions. Bottom panel: relative percent error on the output voltage.

The top panel of Figure 13 shows the measured (black) and estimated (red) output voltage at electrical steady-state for all the considered operating conditions. The relative percent error is shown in the bottom panel. Thanks to the correction term η, the two curves almost overlap (the error was always below 1%).

## 4. Discussion

The proposed observer achieved a relative percent error below 10% in estimating the current ripple at steady-state and during the slow thermal transients, also when the inductor worked in saturation and observers based on the classical linear inductor would fail. The accuracy could be considered satisfactory, considering that it was coherent with the inductor model accuracy [20] and that the inductance tolerance of the considered components was 30%. Offline results during fast electrical transients showed that the estimated current and voltage profile was qualitatively similar to the measured one. The delays in reaching the convergence, due to model inaccuracies, were lower than 2 ms in the considered scenarios. This suggested that the proposed observer could be possibly exploited for current mode control of SMPS or for more sophisticate model-based control strategies such as model predictive control [26]. The main drawback of this approach was the computational effort, which was strongly reduced with respect to [21], due to the analytical formulation, but was still high for a low-cost microcontroller (e.g., the STM32F4 exploited in this work). The latency of 200 μs, indeed, prevented the observer from being coupled with a controller, due to the high delays during electrical transients. To this aim, a high-performance microcontroller, an SoC, or an FPGA should be used. However, the proposed implementation could still be exploited for monitoring purposes, in order to sense, for example, that the inductor current is increasing due to a change in the operating conditions or due to the slow thermal drift. To this aim, indeed, it is only necessary to acknowledge quickly when the current overshoots a safety threshold without accurately reproducing the current profile. This task can be accomplished by the proposed implementation in a few milliseconds.

## 5. Conclusions

In this paper, we proposed an analytical formulation of an observer, able to estimate the current ripple in saturating inductors within switch-mode power supplies. A recently proposed PWA inductance model was exploited, which took into account the magnetic saturation and the dependence of the inductance on the core temperature. Tests were performed both during fast electrical transients and slow thermal transients on a real boost converter, where the observer was implemented on a low-cost microcontroller. A disturbance term was also estimated, which allowed partially compensating for the model parameters’ inaccuracies. With the selected microcontroller, the observer had satisfactory performances during slow thermal transients and at steady-state. The observer could therefore be exploited, e.g., for monitoring of the SMPS. In order to exploit the proposed observer for current mode control, a faster hardware must be used, which is the subject of future research.

## Figures and Tables

**Table 1:** Operating conditions used for training the models.

Parameter	Values
V	5.5 V
I	1.2,1.4,1.6,1.8,2,2.2 A
F	70 kHz
D	0.4,0.5

**Table 2:** Models’ parameters.

Name	Value	Name	Value
α	$-4.87\cdot 10^{-1}$ A/W	β	5.25 A
γ	$2.13\cdot 10^{-2}$ Ω	δ	$1.15\cdot 10^{-1}\Omega$
$R_{L}$	35 mΩ	τ	85.50 s
$L\left(X_{0}\right),\ldots ,L\left(X_{m}\right)$	12.1189 μH, 12.0834 μH, 12.0312 μH, 11.9543 μH, 11.8027 μH, 11.5529 μH, 10.3413 μH,		
	2.9057 μH, 1.6942 μH, 1.4444 μH, 1.2927 μH, 1.2158 μH, 1.1638 μH, 1.1274 μH		

**Table 3:** Switch-mode power supply (SMPS) operating conditions during transient tests.

#	*V*	*I*	*F*	*D*
#1	0V → 5.5V	1.4A	70kHz	0.5
#2	5.5V	1A → 2.5A	70kHz	0.5
#3	5.5V	2.5A → 1A	70kHz	0.5
#4	5.5V	2A	100kHz → 50kHz	0.5
#5	5.5V	1.5A	70kHz	0.4 → 0.5

**Figure A1:**
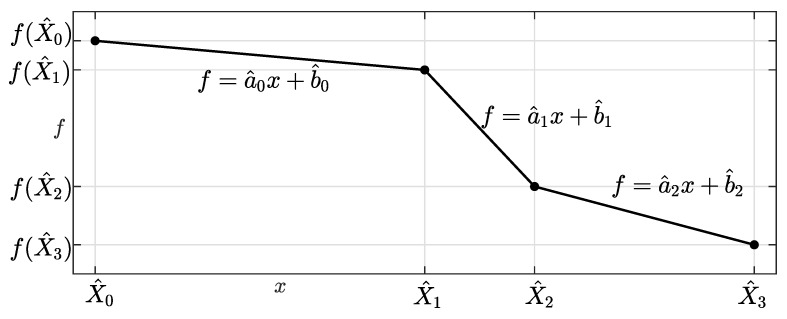
Example of the piecewise-affine (PWA) function f(x) with m=3.

**Figure A2:**
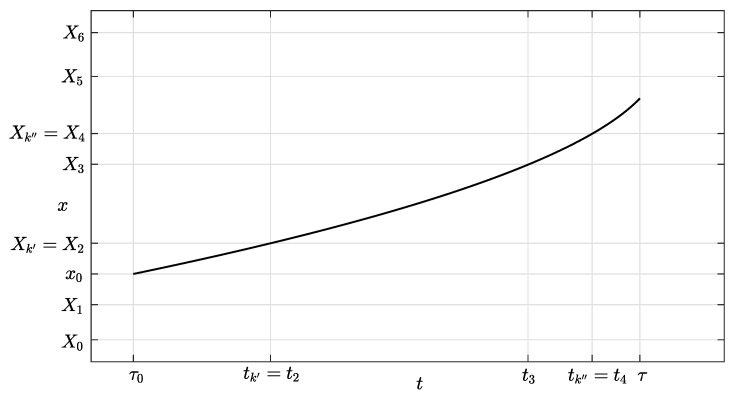
Example of function x(t), with m=6, $k^{\prime }=2$, and $k^{\prime \prime }=4$.

## Appendix A

Consider a continuous PWA function $f\left(x\right):D\to R^{+}$, with $D=\{x\in R:{\hat{X}}_{0}\leq x\leq {\hat{X}}_{m}\}$ and knee points ${\hat{X}}_{k}$, k=1,…,m−1 (see Figure A1), such that ${\hat{X}}_{k}<{\hat{X}}_{k+1}$, k=0,…,m−1. We remark that f(x)>0, ∀x∈D. This function can be defined as:

(A1) $$f\left(x\right)={\hat{a}}_{k}x+{\hat{b}}_{k},\;\mathrm{if}{\hat{X}}_{k}\leq x<{\hat{X}}_{k+1},\;k=0,\ldots ,m-1$$

Consider the following initial value problem:

(A2) $$\left\{\begin{array}{c} \frac{dy}{dt}=\frac{W}{f(y-\bar{y})}\\ y\left(\tau_{0}\right)=y_{0} \end{array}\right.$$

where ${\hat{X}}_{0}+\bar{y}\leq y\leq {\hat{X}}_{m}+\bar{y}$ and *W* and $\bar{y}$ are constant parameters. We are interested in analytically computing y(τ), $\int_{\tau_{0}}^{\tau }y\left(t\right)dt$ and $\int_{\tau_{0}}^{\tau }y^{2}\left(t\right)dt$, as a function of parameters *W*, $y_{0}$, $\bar{y}$, $\tau_{0}$, and τ.

By defining:

(A3) $$x≜y-\bar{y},$$

Problem (A2) can be redefined as:

(A4) $$\left\{\begin{array}{c} \frac{dx}{dt}=\frac{W}{f(x)}\\ x\left(\tau_{0}\right)=y_{0}-\bar{y}≜x_{0} \end{array}\right.$$

We assume that *y* and $\bar{y}$ are such that ${\hat{X}}_{0}\leq x\left(t\right)\leq {\hat{X}}_{m}$, $\forall t\in [\tau_{0},\;\tau ]$.

Notice that, since f(x)>0, x(t) is monotonically increasing if W>0 and monotonically decreasing if W<0. We define variables $X_{k}$, k=0,…,m as:

(A5) $$X_{k}=\left\{\begin{array}{c} {\hat{X}}_{k},\;\mathrm{if}W>0\\ {\hat{X}}_{m-k},\;\mathrm{if}W<0 \end{array}\right.$$

We also define variables $a_{k}$ and $b_{k}$, k=0,…,m−1, as:

(A6) $$a_{k}=\left\{\begin{array}{c} {\hat{a}}_{k},\;\mathrm{if}W>0\\ {\hat{a}}_{m-k-1},\;\mathrm{if}W<0 \end{array}\right.\;b_{k}=\left\{\begin{array}{c} {\hat{b}}_{k},\;\mathrm{if}W>0\\ {\hat{b}}_{m-k-1},\;\mathrm{if}W<0 \end{array}\right.$$

Figure A2 shows a possible time evolution of x(t) for W>0 (the case W<0 is analogous, mutatis mutandis), starting at $x_{0}$ for $t=\tau_{0}$ and ending at x(τ) for t=τ. Variables $X_{k}$ are indicated on the vertical axis. We denote as $t_{k}$ the time instants such that $x\left(t_{k}\right)=X_{k}$, k=0,…,m.

In order to compute the desired quantities, it is necessary to identify the linear regions of the PWA function shown in Figure A1 containing $x_{0}$ and x(τ). To this aim, we denote with $k^{\prime }$ the index *k* such that $x_{0}\in \left[X_{k^{\prime }-1}\;X_{k^{\prime }}\right)$ and with $k^{\prime \prime }$ the index *k* such that $t_{k^{\prime \prime }}<\tau <t_{k^{\prime \prime }+1}$. The identification of $k^{\prime }$ is faster if knee points are equally spaced within the domain. It may also happen that $t_{k^{\prime }-1}<\tau <t_{k^{\prime }}$ (i.e., both $x_{0}$ and x(τ) lie in the same region), which implies that $k^{\prime \prime }=k^{\prime }-1$. The following sections are referred to the most common (and complex) case, where $k^{\prime \prime }\neq k^{\prime }-1$. The case $k^{\prime \prime }=k^{\prime }-1$ is discussed in Appendix A.4.

### Appendix A.1. Computation of x(t)

Since $X_{k^{\prime \prime }}$ is known (it is a knee point), Problem (A4) can be recast as:

(A7) $$\left\{\begin{array}{c} \frac{dx}{dt}=\frac{W}{f(x)}\\ x\left(t_{k^{\prime \prime }}\right)=X_{k^{\prime \prime }} \end{array}\right.$$

The solution of this problem, however, requires computing time $t_{k^{\prime \prime }}$, which can be evaluated recursively starting from time $t_{k^{\prime }}$. Time $t_{k^{\prime }}$ can be computed by integrating both sides of Equation (A4) between $\tau_{0}$ and $t_{k^{\prime }}$, which leads to:

(A8)∫x0Xk′f(x)dx=∫τ0tk′Wdt(A9)ak′−12(Xk′2−x02)+bk′−1(Xk′−x0)=(tk′−τ0)W

We can then obtain $t_{k^{\prime }}$ as:

(A10) $$t_{k^{\prime }}=\tau_{0}+\frac{a_{k^{\prime }-1}X_{k^{\prime }}^{2}+2b_{k^{\prime }-1}X_{k^{\prime }}-a_{k^{\prime }-1}x_{0}^{2}-2b_{k^{\prime }-1}x_{0}}{2W}$$

If we now integrate both sides of Equation (A4) between $t_{k-1}$ and $t_{k}$, with $k^{\prime }<k\leq k^{\prime \prime }$, we obtain:

(A11) $$t_{k}=t_{k-1}+\frac{a_{k-1}X_{k}^{2}+2b_{k-1}X_{k}-a_{k-1}X_{k-1}^{2}-2b_{k-1}X_{k-1}}{2W}=t_{k-1}+\frac{\Phi_{k-1}}{W}$$

where terms $\Phi_{k}≜\frac{1}{2}\left(a_{k}X_{k+1}^{2}+2b_{k}X_{k+1}-a_{k}X_{k}^{2}-2b_{k}X_{k}\right)$ can be computed offline, for k=0,…,m−1, as they only depend on function *f*. It also appears that $\Phi_{k}=W\left(t_{k+1}-t_{k}\right)$.

Once time $t_{k^{\prime \prime }}$ has been obtained (by recursively applying (A11)), it is possible to solve Problem (A7) by integrating between $t_{k^{\prime \prime }}$ and τ, thus obtaining:

(A12) $$x\left(\tau \right)=\frac{-2b_{k^{\prime \prime }}+\sqrt{4b_{k^{\prime \prime }}^{2}+4a_{k^{\prime \prime }}\left[a_{k^{\prime \prime }}X_{k^{\prime \prime }}^{2}+2b_{k^{\prime \prime }}X_{k^{\prime \prime }}+2\left(\tau -t_{k^{\prime \prime }}\right)W\right]}}{2a_{k^{\prime \prime }}}$$

### Appendix A.2. Computation of $\int_{\tau_{0}}^{\tau }x(t)dt$

By rewriting Equation (A12) for a generic $k\geq k^{\prime }$ and $t\in [t_{k},\;t_{k+1})$, it is possible to recast it as:

(A13) $$x\left(t\right)=A_{k}+B_{k}\sqrt{C_{k}+D_{k}\left(t-t_{k}\right)W}$$

with $A_{k}=-\frac{b_{k}}{a_{k}}$, $B_{k}=\frac{1}{2a_{k}}$, $C_{k}=4b_{k}^{2}+4a_{k}\left(a_{k}X_{k}^{2}+2b_{k}X_{k}\right)$, and $D_{k}=8a_{k}$. Terms $A_{k}$, $B_{k}$, $C_{k}$, and $D_{k}$ can be computed offline for k=0,…,m−1, as they only depend on function *f*. For $k=k^{\prime }-1$ and $t\in [t_{k^{\prime }-1},\;t_{k^{\prime }})$, x(t) is obtained as:

(A14) $$x\left(t\right)=A_{k^{\prime }-1}+B_{k^{\prime }-1}\sqrt{{\hat{C}}_{k^{\prime }-1}+D_{k^{\prime }-1}\left(t-\tau_{0}\right)W}$$

where ${\hat{C}}_{k^{\prime }-1}=4b_{k^{\prime }-1}^{2}+4a_{k^{\prime }-1}\left(a_{k^{\prime }-1}x_{0}^{2}+2b_{k^{\prime }-1}x_{0}\right)$, which depends on the initial condition $x_{0}$.

The requested integral can be split into different intervals:

(A15) $$\int_{\tau_{0}}^{\tau }x\left(t\right)dt=\int_{\tau_{0}}^{t_{k^{\prime }}}x\left(t\right)dt+\sum_{k^{\prime }\leq k<k^{\prime \prime }}\int_{t_{k}}^{t_{k+1}}x\left(t\right)dt+\int_{t_{k^{\prime \prime }}}^{\tau }x\left(t\right)dt$$

The first integral depends on *W* and $x_{0}$ and can be analytically computed by using the expression of x(t) in (A14). The last integral depends on *W* and τ and can be analytically computed by using the expression in (A12) by replacing τ with *t*. The remaining integrals only depend on *W* and can be analytically computed by using the expression of x(t) in (A13). It is easy to verify that:

(A16) $$\int_{t_{k}}^{t_{k+1}}x\left(t\right)dt≜\frac{I_{k}}{W}$$

where terms $I_{k}$ only depend on function *f* and can be therefore computed offline for k=0,…,m−1.

### Appendix A.3. Computation of $\int_{\tau_{0}}^{\tau }x^{2}(t)dt$

Furthermore, this integral can be split into different intervals:

(A17) $$\int_{\tau_{0}}^{\tau }x^{2}\left(t\right)dt=\int_{\tau_{0}}^{t_{k^{\prime }}}x^{2}\left(t\right)dt+\sum_{k^{\prime }\leq k<k^{\prime \prime }}\int_{t_{k}}^{t_{k+1}}x^{2}\left(t\right)dt+\int_{t_{k^{\prime \prime }}}^{\tau }x^{2}\left(t\right)dt$$

Furthermore, these integrals can be computed analytically, based on the definition of x(t) provided in Equations (A12)–(A14). It is easy to verify that it is possible to write:

(A18) $$\int_{t_{k}}^{t_{k+1}}x^{2}\left(t\right)dt≜\frac{Q_{k}}{W}$$

where terms $Q_{k}$ only depend on function *f* and can be therefore computed offline for k=0,…,m−1.

### Appendix A.4. Case $k^{\prime \prime }=k^{\prime }-1$

If $k^{\prime \prime }=k^{\prime }-1$, then both x(0) and x(τ) are within interval $\left[X_{k^{\prime \prime }}\;X_{k^{\prime \prime }+1}\right)$. In this case, $f\left(x\right)=a_{k^{\prime \prime }}x+b_{k^{\prime \prime }}$, for $t\in [\tau_{0},\;\tau ]$. Therefore, it is easy to verify that:

(A19) $$x\left(\tau \right)=\frac{-2b_{k^{\prime \prime }}+\sqrt{4b_{k^{\prime \prime }}^{2}+4a_{k^{\prime \prime }}\left(a_{k^{\prime \prime }}x_{0}^{2}+2b_{k^{\prime \prime }}x_{0}+2\left(\tau -\tau_{0}\right)W\right)}}{2a_{k^{\prime \prime }}}$$

The integrals between $\tau_{0}$ and τ of x(t) and $x^{2}\left(t\right)$ can be computed analytically, based on the definition of x(t) in Equation (A19).

### Appendix A.5. Computation of y(τ), $\int_{\tau_{0}}^{\tau }y\left(t\right)dt$ and $\int_{\tau_{0}}^{\tau }y^{2}\left(t\right)dt$

By applying Equation (A3), we can compute:

(A20) $$y\left(\tau \right)=x\left(\tau \right)+\bar{y}$$

(A21) $$\int_{\tau_{0}}^{\tau }y\left(t\right)dt=\int_{\tau_{0}}^{\tau }\left[x\left(t\right)+\bar{y}\right]dt=\int_{\tau_{0}}^{\tau }x\left(t\right)dt+\left(\tau -\tau_{0}\right)\bar{y}$$

(A22) $$\int_{\tau_{0}}^{\tau }y^{2}\left(t\right)dt=\int_{\tau_{0}}^{\tau }{\left[x\left(t\right)+\bar{y}\right]}^{2}dt=\int_{\tau_{0}}^{\tau }x^{2}\left(t\right)dt+2\bar{y}\int_{\tau_{0}}^{\tau }x\left(t\right)dt+\left(\tau -\tau_{0}\right){\bar{y}}^{2}$$

where all quantities are known.

### Appendix A.6. Remarks

The evaluation of y(τ), $\int_{\tau_{0}}^{\tau }y\left(t\right)dt$ and $\int_{\tau_{0}}^{\tau }y^{2}\left(t\right)dt$ must be performed many times, during the estimator operation, with different values of $y_{0}$, $\bar{y}$, τ, $\tau_{0}$, and *W*. However, all terms $A_{k}$, $B_{k}$, $C_{k}$, $D_{k}$, $\Phi_{k}$, $I_{k}$, and $Q_{k}$ can be computed only once offline, as they only depend on function *f*, which does not change. This allows sparing many online computations, thus reducing the circuit latency.
